# Debating: effective and satisfactory learning method in dentistry

**DOI:** 10.1186/s12909-024-05286-5

**Published:** 2024-03-19

**Authors:** Marjaneh Meschi, Samane Shirahmadi, Mahrokh Amiri, Nikki Ebrahimi-Siaghi

**Affiliations:** 1 Department of Community Oral Health, School of Dentistry and Dental Research Centers, Hamadan University of Medical Sciences, Hamadan, Iran; 2 Department of Community Oral Health, School of Dentistry, Hamadan University of Medical Sciences, Hamadan, Iran; 3 Student of Biology, Faculty of Science, University of British Columbia, Vancouver, BC Canada

**Keywords:** Dentistry, Debating, Active learning, Students' perceptions, Critical thinking skills, Reasoning skills

## Abstract

**Background:**

Education in the modern world of health needs diverse methods of learning and teaching. The traditional education model has limited capacity for developing abilities such as critical thinking, problem-solving, and reasoning skills. Therefore, improving the quality of teaching–learning processes requires implementing educational innovations in the classroom and evaluating them. This study aimed to determine the impact of the debate teaching method on improving the abilities of general dentistry doctoral students.

**Methods:**

The research was a semi-experimental study with pre-tests and post-tests to measure the knowledge and abilities of students. The study included 60 dental students who completed the fall 2022 session of the Community Oral Health (COH) 2 practical course. This course, one of three practical components within the Community Oral Health curriculum, aligns with the educational framework of general dentistry. Challenging topics on which there is no consensus in dentistry were chosen for the debate. The descriptive statistics indicators include an independent t-test and variance analysis test with a significance level of 5%. Were used to analyze the data.

**Results:**

The results of the study showed that the average total knowledge (*P* < 0.001), 'perception of critical thinking skills (*P* < 0.001), expression power (*P* < 0.001), reasoning skills (*P* = 0.003), interpretation and Information analysis power (*P* < 0.001), the ability to find and use scientific databases (*P* < 0.001) and the ability to analyze and evaluate evidence (*P* < 0.001) increased significantly after intervention in students. 95% of students agreed/strongly agreed that this method enhances their ability to answer people's questions. From an instructor’s point of view, students had 93.1% of the ability to reason and analyze information after intervention and 88.5% of the ability to think critically.

**Conclusion:**

The results of the study showed that the use of debate in the classroom is an effective way to present content. The process of evaluating data-driven arguments promotes higher-level cognitive skills and teaches students about the knowledge base and the use of scientific databases.

**Trial registration:**

Registration date: 21/11/2022, Registration number: IRCT20141128020129N3.

**Supplementary Information:**

The online version contains supplementary material available at 10.1186/s12909-024-05286-5.

## Background

In the past thirty years, fundamental changes have been made in the effective methods of learning and teaching, and the enhancement of abilities such as critical thinking, problem-solving, and reasoning have been considered as the main goals of learning and teaching [1]. One of the new learning theories that strengthen these skills in learners is constructivism, which emphasizes the active participation of learners in various aspects of the subject being taught. Constructivism is based on participatory and exploratory learning. In this method, group members have the opportunity to share their opinions and come to a consensus on the discussed ideas. Therefore, learners not only contemplate their own perspectives but also review and examine the opinions of their peers [2]. On the other hand, in this method, learners critically evaluate and discuss various information pertaining to the teaching subject. This critical insight leads to a profound comprehension of the subject [2, 3]. While it is commonly assumed that the educational curriculum for each discipline aims to improve the scientific and professional level of learners in their respective fields in order to nurture their analytical, argumentative, and problem-solving skills- skills that are well developed through constructivism, constructivist teaching in scientific lectures and theoretical science teaching remains less used. Usually, teaching in universities is through traditional lecturing [4].

Constructivist teaching tools, such as case studies and problem-based learning strategies, are well-known approaches for developing interaction in the classroom, enhancing skills such as critical thinking and analytical abilities, and improving learners' social skills and participation, as well as enhancing their knowledge retention [5]. The real challenge or perceived difficulty of employing these tools, and the amount of time required to implement them are obstacles to the administration of these strategies [1]. To overcome these obstacles, alternative constructivist teaching methods are needed that can effectively present the content, be easy to use, and be compatible with a particular course. Debate is one of these methods [4]. Recent studies describe its application in diverse fields [4, 6–8]. By engaging in this method, learners are encouraged to analyze, synthesize, and evaluate ideas through resource evaluation, appraisal of resource appropriateness, searching for connections in data sets, and examining different perspectives [9].

Studies reveal that dentistry is a dynamic and complex clinical field and having abilities such as critical thinking, problem-solving, scientific reasoning, and information analysis in heterogeneous groups and complex environments is essential for applying theoretical knowledge to professional practice [10].

On the other hand, there are many subjects in dentistry that have a complex nature and are constantly changing and, despite extensive studies, there is no consensus on them. One of the approaches to learn these challenging topics for dentists is debating [8]. Debate is a method in which the topic is analyzed by all learners, ideas are shaped through resource evaluation, and different points of view are heard and evaluated. However, multiple studies have shown that the traditional model of information transfer by traditional teaching models commonly used in most dental schools has a limited capacity for developing these skills [11, 12]. This issue highlights the need for exploring alternative methods of organization of university-level teaching. Research findings in education over the past two decades, aimed at addressing the shortcomings of traditional teaching approaches, demonstrate the influential role of debate in developing these skills [13, 14].

The study by Rubin in 2008 addresses the limited application and evaluation of debates in dental education, despite the complexity and dynamic nature of scientific dentistry. Rubin's research found that debates were an effective method for improving dental students' knowledge and engagement. However, the study primarily relied on student feedback for its conclusions, indicating a potential gap in measuring the actual depth of learning through this educational method [8].

Since improving the quality of teaching–learning processes requires the implementation of educational innovations in the classroom and their evaluation, this study was conducted with the aim of implementing and evaluating the debate teaching method to 11th semester students of the general dentistry doctoral program as part of the oral health and community dentistry course.

## Methods

### Participants

In this study, a semi-experimental design with pre-test and post-test was employed to measure the improvement of students' abilities. Sixty dental students in their 11th semester at Hamadan University of Medical Sciences (located in western Iran) participated in this study during the autumn of 2022. The participants were selected using a census sampling method, and all the students who had taken course of Practical Oral Health and Community Dentistry 2in the first semester of the academic year 2022–2023 were chosen as the target group for the study.

The Department of Community Oral Health within the Faculty of Dentistry offers three practical courses (Practical Community Oral Health 1, Practical COH2, and Practical COH3) alongside two theoretical courses. These courses aim to enhance knowledge, foster attitudinal shifts, enhance student performance in the realms of oral and dental disease prevention and promotion, and ultimately, elevate individuals' quality of life. Throughout the educational program, achieving these objectives students involve in social activities and staying abreast of the latest scientific evidence. Practical COH2 specifically emphasizes mastering principles of evidence-based dentistry, enhancing critical thinking and reasoning, analyzing information, and improving the ability to present subjects among students.

Considering that there are many topics in dentistry that are important in terms of public health, policy, and culture, and have been extensively discussed and studied in both academic and governmental circles, it is still noteworthy that there is no consensus on these subjects. Therefore, these topics have been chosen for student debates. These topics include: The use or non-use of water fluoridation for drinking purposes, the use or non-use of amalgam in dental restorations, the use of antibiotics in dentistry, dental treatments of pregnant women, the use or non-use of electronic cigarettes, the use or non-use of fissure sealants in dentistry, and the impact of social and behavioral factors on oral health. The students were provided with scenario-based assignments related to these subjects and completed their debate-related tasks mostly outside the classroom.

Students who took the practical courses of Oral Health and Community Dentistry in the second semester of the academic year 2022–2023 and were willing to participate in the study were included, while students who were assigned a thesis or studies related to the given subject were excluded from the study.

Sample size was calculated based on the following formula: (z1-α/2 + z1- β/2)2 (p1 (1-p1) + p2 (1-p2)) / (p1-p2)2. The values of p_1_ and p_2_ indicate the proportion of knowledge before and after intervention which was estimated based on previous study [4]. The p_1_ and p_2_ were considered 0.56 and 0.3 respectively.

Power of 80%, 95% confidence level and 10% attrition rate was considered. Totally, 53 students were recruited.

### Debating

Before starting the program, students were randomly divided into 7 groups of 8–10 members. The academic faculty members acted as supervisors and coordinators in these sessions.

The debating process consisted of 3 main areas: 1) Preparation 2) Implementation 3) Feedback.

### Preparation

In this stage, the purpose of conducting the debate and the general outline of how the debate should be conducted were explained to each group of students. Clear instructions regarding objectives, purposes, and how to conduct debate were provided to the students. This included information on structure, format, and duration for each stage (scenario introduction, opposition arguments, refutation statements, concluding statements followed by open discussion), as well as evaluation criteria. The students were informed that debating was meant to be a learning experience for them.

Each group worked on the same topic. Each group was responsible for discussing and researching the assigned topic. The students prepared an introduction containing general information about their topic in one week.

At the end of the deadline, instructors asked the groups to evaluate their introductions considering the following questions: Does your collected evidence include basic information about the topic, including its connection to cultural/social issues (such as communities impacted by the subject), economic issues, and health-related concerns? Does your collected evidence include statistics that support this piece of information? If so, how and when were these data collected? How do these factors affect the selection of relevant evidence and your choice to utilize this evidence? The students responded to the questions through discussion with each other (for 15 min). The students were given two extra days to complete their information if necessary.

Following the completion of the introduction, a debate-provoking hypothesis regarding the subject was given to each group of students (Table 1), and each group started collecting evidence in line with this hypothesis in the next stage. The students were given one week to collect arguments for and against the debate-provoking hypothesis and present the supporting evidence for both positions to the instructors. Small group discussions were held in the presence of instructors, and the guides provided feedback on the information collected by the students.

**Table 1:** Debate topics

Topic	Debatable Areas
The use or non-use of amalgam in dental restorations	Mercury content and toxicity, Systemic and oral health effects, Treatment failure, Aggressive preparation, Compared with other restorative materials
The use or non-use of water fluoridation for drinking purposes	systemic and oral health effects, Social justice, Cost-effectiveness
The use of antibiotics in dentistry	systemic and oral health effects of systemic and topical administration, Antibiotic resistance, Indications in dental treatments and Dentists' knowledge, Prescription methods, Cost-effectiveness,
Dental treatments of pregnant women	Medicines, Radiation, Given stress during dentistry Dentists' knowledge
The use or non-use of electronic cigarettes	toxicity, Its' systemic and oral health effects, Compared with conventional cigarettes Advertisements
The use or non-use of fissure sealants in dentistry	Materials, Isolation Failure Cost-effectiveness,
The impact of social and behavioral factors on oral health	The impact of social determinant of health such as general literacy, health literacy, laws The effect of health promoting behaviors such as brushing and flossing

After one week, the students were asked to review and revise their collected evidence, based on the following questions: Does your information include data-based evidence for each situation? Are the data objective or subjective? How the data were collected, and how does the method of data collection affect your decision to use it? Does your information cover all aspects of each situation (including economic issues, health effects, medical effects, cost-effectiveness, and impact on social justice)? The students were asked to bring these materials to the class and present them to the instructors. Small group discussions were conducted in the presence of instructors, and the instructors provided feedback on the information collected by the students.

After two additional days to complete the information, each group was randomly divided into smaller groups (5–4 members). In the other word each group was assigned a position (for or against) regarding the debate-provoking hypothesis, and given one week to complete the final part of the debate. This part included preparing persuasive arguments for each group's position compared to the opponent. Necessary coordination regarding the implementation of the debate was also done by the instructors. After one week, each group individually presented their argument, and small group discussions were held in the presence of instructors, and the instructors provided their feedback.

The debate was conducted with opposing and supporting groups facing each other behind a U-shaped table. The standard debate method [15] was employed and the initiating group for each debate was randomly selected. Then, the introduction of the debate was presented by the first person for 4 min. The next debaters had 3 min each to present their arguments. Each student from each group was responsible for responding to an aspect raised by the opposing group. The debate lasted a maximum of 30 min. At the end, a final summary was presented by the students for 10 min. Simultaneously with the debate, a panel of judges (consisting of the teaching staff providing the module) evaluated the performance of the debaters based on an evaluation checklist (Additional file 1).

### Feedback

The debate process was finalized with feedback. At this stage, tutors provided their opinions to the students regarding the preparation process, the debate itself, and the overall performance of each group. The individual performance of each student in terms of the necessary skills was also discussed with the students. Finally, the students were asked to complete questionnaires prepared by the authors.

### Evaluation

Students' knowledge: The students' knowledge was evaluated through descriptive responses to the questions regarding the provocative hypothesis. The provocative hypotheses that formed the basis of the group debates were designed by the faculty members of the Community Oral Health department, and the validity of the scenarios, questionnaires, and activities and their relationship with the study goals was confirmed by experienced faculty members from the relevant groups.

#### Student’s perceptions of their abilities

The measurement of student’s perceptions of their abilities was collected through a questionnaire.

#### Students' perceptions of the usefulness of the debate

The measurement of student’s perceptions of the usefulness of the debate for enhancing their abilities and capabilities was collected through a questionnaire.

#### Participants’ Skills

The skills and capabilities of the debate team members were evaluated by the tutors throughout the debate using the Skills and Capabilities Assessment Checklist (Additional file 1).

The provocative hypothesis and the questionnaire measuring students' perceptions of their abilities were given to the students immediately after the debate topics were assigned to each group and before the scientific research began. In addition, the questionnaire was given to the students immediately after the debate. The questionnaire to measure the students' perceptions of the usefulness of the debate for enhancing their skills and capabilities was given to the students immediately after the debate. The questionnaires were administered to students under the direct supervision of one of the faculty members.

### Development and Validity

Samples of questionnaires used in studies [16–18] were used to design and construct the questionnaire on students' perceptions of their abilities and the questionnaire on the usefulness of the debate.

The questionnaire on students' perceptions of their abilities included 8 questions on a 5-point Likert scale.

The questionnaire on students' perceptions of the usefulness of the debate for enhancing their skills and capabilities included 9 questions on a 5-point Likert scale. It also included open-ended questions aimed at identifying the benefits of debate, factors that lead to its inefficiency, ways in which debate helps students as experts, and feedback/suggestions for improving debate sessions.

A checklist evaluating students' skills and abilities based on conducted studies was prepared [4, 18].

The validity and reliability (Cronbach's alpha) of the questionnaire on the students' perception of their skills, the questionnaire on the usefulness of the debate and the checklist to assess skills were evaluated. The validity of the questionnaires was confirmed from the perspective of 10 specialists in the field of health education, health promotion and community oral health. The content validity ratio (CVR) and the content validity index (CVI) were calculated. The CVI, CVR and Cronbach's alpha of the questionnaires are shown in Table 2. The face validity of the questionnaires was evaluated by 30 students (10th semester dental students). Their characteristics were similar to the target sample of the study.

**Table 2:** Content validity and internal consistency of the questionnaires

Questionnaires	Cronbach alpha (> 0.7)^a^	CVR	CVI
Students' perceptions of their abilities	0.80	0.84	0.72
Students' perceptions of the usefulness of the debate for enhancing their skills and capabilities	0.90	0.74	0.77
Checklist evaluating students' skills and abilities	-	0.81	0.7

^a^Indicates an acceptable level of reliability or validity [19]

### Statistical analysis

The collected data from the questionnaire were analyzed using SPSS software version 16. Descriptive statistics, including prevalence rates, central tendencies, and dispersion indices, were used. Independent t-test comparing before and after and analysis of variance for comparing more than two groups with a significance level of 5% applied. To facilitate the presentation of the questionnaire measuring students' perceptions of their abilities, the measurement levels were recoded as follows: low and very low = low, high and very high = high.

## Result

**Fig. 1:**
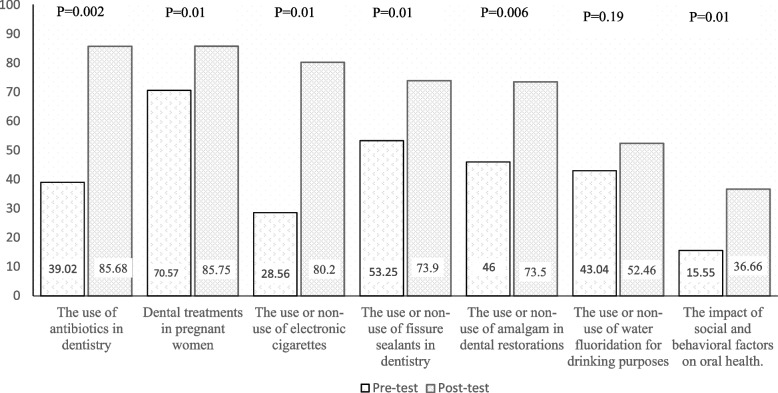
Mean pretest and posttest ratings of knowledge (*N* = 60)

Sixty dental students in their 11th semester participated in this study, of whom 58.3% were male and 41.7% were female. The mean age of participants was 25.36 (± 3.16). The results of this study showed that the students' knowledge pertaining to their respective group topics increased after the intervention. The average knowledge score of the students before the study was 42.5 (± 23.94) and after the intervention, it increased to 67.71 points (± 22.66), and this difference was statistically significant (*P* < 0.001, Fig. 1). The most substantial difference in knowledge scores before and after the intervention was related to the group the use or non-use of electronic cigarettes, with an increase of 51.64 points (Fig. 1).

Furthermore, the results of this study indicated that the students' perception of critical thinking abilities (*P* < 0.001), Presentation ability (*P* < 0.001), reasoning ability (*P* = 0.003), Data analysis ability (*P* < 0.001), ability to find information and scientific databases use (*P* < 0.001), and their ability to analyze primary literature (*P* < 0.001) significantly increased after the intervention (Table 3).

**Table 3:** Participant’s perceptions

Student’s perceptions	Post-test			Post-test			*P* value
	Low N (%)	Neutral N (%)	High N (%)	Low N (%)	Neutral N (%)	High N (%)	
Knowledge of their assigned topic.	21(35.0)	17(28.3)	22(36.7)	1(1.7)	5(8.3)	54(90.0)	< 0.001
Knowledge of topics assigned to other groups.	32(53.3)	21(35)	7(11.7)	4(6.7)	24(40.0)	32(53.3)	< 0.001
Their ability to use health and medicine databases	26(43.3)	14(23.3)	20(33.3)	4(6.7)	6(10.0)	50(83.3)	< 0.001
Their ability to analyze primary literature.	22(36.7)	15(25.0)	23(38.3)	2(3.3)	9(15.0)	49(81.7)	< 0.001
Presentation ability	21(35.0)	10(16.7)	29(48.3)	6(10.0)	11(18.3)	43(71.7)	< 0.001
Reasoning ability	10(16.7)	16(26.7)	34(56.7)	3(5.0)	11(18.3)	46(76.7)	0.003
Critical thinking ability	14(23.3)	23(38.3)	23(38.3)	4(6.7)	10(16.7)	46(76.7)	< 0.001
Data analysis ability	10(16.7)	22(36.7)	28(46.7)	3(5.0)	9(15.0)	48(80.0)	< 0.001

Before the intervention, 43.3% of the students perceived their ability to use health and medicine databases as low, while 33.3% perceived it as high, which after the intervention the scores increased to 76.6% and 83.3% respectively. Additionally, before the intervention, 35% of the students perceived their knowledge of the assigned topic as low and 36.7% perceived it as high, which after the intervention rose to 71.4% and 90% respectively (Table 3).

95% of the students agreed/strongly agreed that debate is better than having class discussion on controversial topic, and the same percentage of them agreed/strongly agreed that debate enhanced their skills to answer questions in front of group of people (Table 4).

**Table 4:** Students’ responses about experience of debate sessions by number and percentage

	Strongly disagreedN (%)	Disagree N (%)	Neutral N (%)	Agree N (%)	Strongly agree N (%)
Better than having class discussion on controversial topic	0(0.0)	0(0.0)	3(5.0)	21(35.0)	36(60.0)
Learned more about controversial topics	0(0.0)	0(0.0)	3(5.0)	23(38.3)	34(56.7)
Good way to explore and research issues	0(0.0)	1(1.7)	7(11.7)	21(35.0)	31(51.7)
Enhanced skills to answer questions in front of group of people	0(0.0)	0(0.0)	3(5.0)	21(35.0)	36(60.0)
Learned how body language influences a person’s perception and decision-making	0(0.0)	1(1.7)	10(16.7)	23(38.3)	26(43.3)
Helped to understand the importance of listening to different viewpoints	0(0.0)	0(0.0)	5(8.3)	26(43.3)	29(48.3)
Improved critical thinking skills	0(0.0)	0(0.0)	6(10.0)	20(33.3)	34(56.7)
Assisted to learn new ways of communication	0(0.0)	2(3.3)	10(16.7)	18(30.0)	30(50.0)
Encouraged to listen to different strategies to convince others	0(0.0)	0(0.0)	6(10.0)	24(40.0)	30(50.0)

In the free text comments, students reported that engaging in the debate helped them in a new way to find differences between issues and make evidence-based decisions. Some participants even mentioned that now they can apply their newfound competence to distinguish between anecdotal information and evidence. Another comment suggested the integration of additional debate sessions within the curricula to optimize efficacy.

Negative experiences identified by the students that hindered learning were related to deficiency in group dynamics, emotional outbursts, insufficient preparation, and being dominant by some participants, all of which sometimes impeded the debate process. Some students complained that the preparation phase of the debate process was too time-consuming.

Reasoning skills had the highest average score of 11.18 ± 1.37, while critical thinking had the lowest average score of 10.63 ± 1.94 (Table 5). According to the tutors' perspective, after the intervention, 93.1% of the students had the ability to reasoning skills, and 88.5% had critical thinking skills.

**Table 5:** Participants’ skill scores

skills	Mean(SD)	Re-range Scores^a^
Reasoning skills	11.18(1.37)	93.1
Data interpretation and analysis skills	11.04(1.33)	92
Ability to use scientific databases	11.16(1.51)	93
Presentation skills	10.92(1.39)	91
Critical thinking skills	10.63(1.94)	88.5

^a^ The scores, re-change to 0–100 for analysis

A review of the students' responses to open-ended questions revealed suggestions for improving the debate process, including implementation of this teaching method across all academic disciplines in their field.

## Discussion

Innovation in teaching/learning methods and evaluating the effectiveness of these approaches has been the subject of many studies in various disciplines for years, however, there have been few studies in the field of dental education [8, 20–22]. This study was conducted with the aim of presenting and evaluating the debate teaching method to dental students in order to improve the quality of the teaching–learning process.

In general, the findings of this study showed that the use of debate in the classroom setting is an effective method for student engagement and enhances the skills needed to cultivate the specialized skills required in the field of dentistry. Students also considered debate as an innovative, interesting, constructive, and useful approach to teaching and learning. The results also showed that debate provides multiple opportunities for developing skills used in scientific research for students. Students engaged in reviewing scientific literature, presenting a summary of a topic, and actively searching for data to support or refute a hypothesis. Studies indicate that lower-level cognitive skills such as the acquisition of knowledge, comprehension and applying information are centered around fragmented learning and memorization, while higher-level cognitive skills such as analysis, synthesis, and evaluation focus on concentrated thinking. Researchers believe that in educating students, the short-term goal of acquiring knowledge should be balanced with the long-term goal of training the mind for analytical and critical thinking [23].

The results obtained from the pre-test and post-test analysis of hypotheses before and after the debate as well as the evaluation of students by tutors during the debate, showed that both lower-level and higher-level cognitive skills improved in students after the intervention. Multiple studies conducted among students in different fields indicate the impact of the debate method on students' knowledge [4, 6, 11]. This result is important and noteworthy in that most of the content is learned independently by students outside the classroom environment, and the learning process is student-centered. Regarding higher-level cognitive skills, in line with other studies conducted in this area [4, 17, 24], the results of this research showed that debate creates a process of information analysis, which helps improve these skills and leads to knowledge construction in students. It is the nature of the debate itself that enables the construction of knowledge and the improvement of higher-level cognitive skills. The reality is that due to the conflicting positions of the students, they had to prepare both favorable and opposing positions in this method. This issue led to a deeper study of the topics. Furthermore, the experience of debate gradually stimulated students to explore deeply in the subject and acquire argumentative skills, as they realized that only traditional approaches to learning do not make them capable of proper defense due to the need to respond to and refute the opinions of the opposing team [6]. Additionally, the debate topics were subjects on which opinions have not unanimous concerns about them so far, and there is no clear consensus in scientific, political, and even general communities regarding them. Therefore, students had to think about different aspects of the topic, organize their thoughts, use reliable sources of information and scientific evidence, and come up with their own answers.

One of the vital elements for enhancing argumentation skills, analysis, and critical thinking, occurred within the process of debate. In this stage, the instructor prepared the students to gradually acquire these skills. While the ultimate responsibility of learning was on the students themselves, teaching by the tutors was an essential part of the debate that aligns with the findings of other authors [6, 25]. The existence of this follow-up process led the students to deeply examine the subject through probing questions, search for arguments and evidence for both positions, and integrate the corrections mentioned by the instructors with their own findings.

The debate brought about balanced participation from all students. This issue not only improved the diligent students but also improved all students with different educational levels in terms of the levels of knowledge, and skills [6]. For this reason, the scores obtained by the students from the debate session, which were given by the instructors, were above 85% for all skills.

The data indicates that the students have gained knowledge and skills, and are able to apply this knowledge and skill. The analysis of the study results, like other similar studies [4], reveals that the students comprehend the increase in knowledge in the field of acquiring and analyzing primary literature, the advance of higher-level cognitive skills (argumentation, analysis, and critical thinking), and improvement in their own presentation style. While there is concern that these results are somewhat more subjective than the results of student assessments, studies show that self-efficacy, belief or individual judgment that one can succeed in a task, increases problem-solving efficiency and therefore, these perceptions play an important role in constructing experience and expertise in the field for each individual [26, 27].

The results obtained from the analysis of students' experiences in participating in debates showed that, like other studies [6, 18, 24, 28], the students believed that participating in classroom debates helped them overcome the fear of speaking in front of an audience, strengthen their self-confidence to speak and express their opinions, and respond to opposing views, improve their speaking skills, and enhance their critical thinking skills. Actually, most of the courses in academic environments are presented in a lecture-based approach and students do not have interactive interactions with their classmates and professors [29]. However, in debates, students found the opportunity to freely express their opinions, speak without anxiety, and enhance their speaking and oral communication skills. Some even stated that they had never spoken and debated in front of a group like this before. For this reason, the participants were very satisfied with the debate learning strategy.

On the one hand, considering that debate involves persuasive arguments, it not only enhances the speaking abilities and skills of the students, but also requires students to actively listen to the perspectives of the opposing groups in order to effectively refute those perspectives. Therefore, in addition to improving speaking abilities, debate also improves students' listening skills and tolerance for opposing viewpoints. In this study, 90% of students claimed that debate encouraged them to listen deeply in order to effectively persuade others, and 91% claimed that debate helped them understand the importance of listening to different viewpoints. These results are consistent with similar studies [18, 30–32].

The study results revealed that debate is very helpful in eliminating biases and discovering issues. In this study, a high percentage of students (71.44%) lacked accurate information about the detrimental effects of electronic cigarettes. A significant proportion of the students with the predetermined idea that electronic cigarettes do not affect oral and dental health, leads to the recommendation of these cigarettes by dentists and the increasing prevalence of these cigarettes among different groups in society. Additionally, before the debate, a high percentage of students (84.45%) believed that the cause of oral and dental diseases in individuals was their failure to adhere to hygiene principles. This predetermined thinking leads to “victim blaming” and causes patients to be criticized by dentists. According to the results of this study, debate was able to eliminate these biases among these students. However, the continuation of these previous perceptions and the usefulness of debate in eradicating them should be examined in future research.

Similar to the results of other studies [17, 24, 30, 31, 33] concerning the impact of debate on increasing argumentation and critical thinking, the outcomes of this study also indicated that engaging in debate leads to increased comprehension of challenging topics and can be an appropriate method for exploring and investigating issues, as well as enhancing critical thinking of students. The rationale for the positive impact of the debate on the improvement of students' abilities was that the participating students in this study were forced to look at a challenging topic from a different perspective for the first time. Therefore, to achieve a proper understanding of the topic, they needed to have logical arguments, search for scientific information and evidence, and effectively use the acquired information to express their opinions to a third party. The debate provided an opportunity for students to go beyond the level of "direct learning of facts, theories, and techniques" to the level of integrating and applying knowledge in a variety of situations and conditions [24, 34, 35]. In this process, students were forced to search for evidence and reasons to support their arguments, look at issues from different angles, and consider multiple perspectives to obtain a deeper understanding and greater mastery of the subject [36]. Going through this process led to the enhancement of critical thinking and problem-solving skills in them.

Another important point of participating in debate was that students were consciously challenged with materials that they completely agreed with but had to play the opposing role in the debate. This allowed students to look at the topic from a different view without bias.

Results of this research showed that applying debate method has remarkable effect on students’ knowledge, critical thinking ability, expression power, reasoning skills, information analysis abilities, and research skills. Both students and instructors considered the debate an effective method in improving learning outcomes and higher-level cognitive abilities.

The results of this study motivate educators to adopt creative teaching methods like debates to improve critical thinking and problem-solving skills among learners. Students can benefit from skill development, self-confidence building, and self-evaluation through involvement in debates. These results also have indication for educational practices and policies, the curriculum revision, assessment strategies, professional development for educators, and promoting student-centered learning approaches. Generally, the study emphasizes the strength of debates in improving student abilities and demands integrating innovative teaching methods to increase and improve the educational experience.

This study also had limitations. Some students did not find speaking and presenting in a group useful, and some disagreed with preparing to defend positions contrary to their own. To overcome these limitations, tutors emphasized the fact that individuals need to step out of their comfort zone for learning to occur [37]. Another limitation was the lack of a follow-up stage. Students graduated at the end of the semester, and access to them was not possible, so the post-test was immediately conducted after the debate. It should also be noted that students' inclination to provide a desirable report of their classroom experiences may affect the reported satisfaction of the debates (acquiescence bias). To overcome this limitation, all questionnaires were collected anonymously by someone outside the research team. Another limitation was the assessment of students' skills based on instructors personal opinions and using a checklist, without a standardized questionnaire to measure critical thinking, argumentation, and active listening skills before and after the intervention. However, all the tutors had completed relevant training courses on critical thinking, argumentation, and active listening and were able to assess the presence or absence of these skills in students. Another limitation of the study was the lack of control groups. It is recommended that future studies include control groups, including groups that have not received any additional training and those who have participated in teaching on the same topic using a fixed method such as lectures or flipped classrooms.

Some of the strengths of the study include preparing two opposing positions for a closed question and examining the collected documents by students supervised by mentors. When students engage in acquiring knowledge, they have the opportunity to make incorrect evaluations or conclusions based on their findings. Tutor feedback is necessary to ensure proper student learning. In this study, after data collection by student groups, small group discussions were conducted with the presence of mentors, allowing mentors to express their opinions on the topics covered during the debate.

## Conclusions

While in the past, the curriculum was a study program that only provided knowledge and then evaluated the students' absorption of that knowledge, now the curriculum should be a collection of experiences in which students encounter information and make judgments about what is important, and use the perspectives they have acquired to understand beliefs and take informed action. Debate is one of the methods that can help students in this process.

The results of this study show that utilizing the debate in the classroom setting is an effective method for presenting the content. The evaluation process of data-driven reasoning enhances higher-level cognitive skills and teaches students how to use scientific databases. Each of these is important in developing expertise in the field. Debate also increases individuals' abilities in critical thinking, analysis, presenting arguments and evidence, and applying all of these in responses. Additionally, it helps individuals develop public speaking skills intuitively and tolerate different opinions and viewpoints.

Although the feasibility of teaching design and possible outcomes may vary in different areas, based on the positive results of this study, the authors urge modern educators to use debate as a teaching method alongside other methods.

### Supplementary Information

**Supplementary Material 1.**

## Data Availability

The author confirms that all data generated or analyses during this study are included in this published article and its supplementary information file.

## References

[1] Tytler R (2002). Teaching for understanding in science: constructivist/conceptual change teaching approaches. Aust Sci Teach J.

[2] Richardson V (2003). Constructivist pedagogy. Teach Coll Rec.

[3] Yarmohammadi E, Jazayeri M, Khamverdi Z, Kasraei S, Rezaei-soufi L (2013). Evaluation of the importance of effective teaching method indicators from dental students' prospects. Avicenna J Dent Res.

[4] Boucaud DW, Nabel M, Eggers CH (2013). Oxford-style debates in a microbiology course for majors: a method for delivering content and engaging critical thinking skills. J Microb Biol Educ.

[5] Barnett J, Hodson D (2001). Pedagogical context knowledge: toward a fuller understanding of what good science teachers know. Sci Educ.

[6] Arrue M, Unanue S, Merida D (2017). Guided university debate: Effect of a new teaching-learning strategy for undergraduate nursing students. Nurse Educ Today.

[7] Kennedy RR (2009). The power of in-class debates. Act Learn High Educ.

[8] Rubin RW, Weyant RJ, Trovato CA (2008). Utilizing debates as an instructional tool for dental students. J Dent Educ.

[9] Hall D (2011). Debate: Innovative teaching to enhance critical thinking and communication skills in healthcare professionals. Internet J Allied Health Sci Pract.

[10] Besimo CE, Zitzmann NU, Joda T (2020). Digital oral medicine for the elderly. Int J Environ Res Public Health.

[11] Alghamdi Hamdan AK, Aldossari AT (2021). Debate learning strategy in female postgraduate school: a Saudi case study. Issues in Educational Research.

[12] Taheri J, Khalighi H, Azimi S, Mortazavi H, Noormohammadi H, Tarahomi M (2012). Oral health knowledge of diabetic patients before and after the education package. J Dent Res.

[13] Mercer N, Littleton K. Dialogue and the development of children's thinking: A sociocultural approach. 1st ed. London and New York: Routledge Taylor & Francis Group Press; 2007.

[14] Osborne J (2010). Arguing to learn in science: the role of collaborative, critical discourse. Science.

[15] Najafi M, Motaghi Z, Nasrabadi HB, Heshi KN (2016). " Debate" Learning Method and Its Implications for the Formal Education System. Educ Res Rev.

[16] Lin S-J, Crawford SY (2007). An online debate series for first-year pharmacy students. Am J Pharm Educ.

[17] Lampkin SJ, Collins C, Danison R, Lewis M (2015). Active learning through a debate series in a first-year pharmacy self-care course. Am J Pharm Educ.

[18] Mumtaz S, Latif R (2017). Learning through debate during problem-based learning: an active learning strategy. Adv Physiol Educ.

[19] Hair J, Anderson R, Tatham R, Black W (1998). Multivariate Data Analysis.

[20] Khan SA, Omar H, Babar MG, Toh CG (2012). Utilization of debate as an educational tool to learn health economics for dental students in Malaysia. J Dent Educ.

[21] Qutieshat A, Maragha T, Abusamak M, Eldik OR (2019). Debate as an adjunct tool in teaching undergraduate dental students. Med Sci Educ.

[22] Shingaki R, Kamioka H, Irie M, Nishimura F (2006). Implementation and evaluation of the debate-style tutorial study in a third-year dental curriculum in Japan. Int Electron J.

[23] Doody O, Condon M (2012). Increasing student involvement and learning through using debate as an assessment. Nurse Educ Pract.

[24] Zare P, Othman M (2015). Students' perceptions toward using classroom debate to develop critical thinking and oral communication ability. Asian Soc Sci.

[25] Tuvesson H, Borglin G. The challenge of giving written thesis feedback to nursing students. Nurse Educ Today. 2014;34(11):1343–5. 10.1016/j.nedt.2014.07.003 25042741

[26] Bandura A (1977). Self-efficacy: toward a unifying theory of behavioral change. Psychol Rev.

[27] Hoffman B, Schraw G (2009). The influence of self-efficacy and working memory capacity on problem-solving efficiency. Learn Individ Differ.

[28] Keynejad RC, Creed S, Fernando M, Bell D, Codling D, Crowther G (2017). Docbate: a national medical student debate. Acad Psychiatry.

[29] Khami MR, Keshavarz H, Razeghi S (2017). Evaluation of last-year dental students' opinions about undergraduate curriculum: before the revision (2010–11). J Dent Med.

[30] Kennedy R. In-class debates: Fertile ground for active learning and the cultivation of critical thinking and oral communication skills. Int J Teach Learn Higher Educ. 2007;19(2):183–90.

[31] Latif R, Mumtaz S, Mumtaz R, Hussain A (2018). A comparison of debate and role play in enhancing critical thinking and communication skills of medical students during problem based learning. Biochem Mol Biol Educ.

[32] Choi YK, Kim EJ (2022). A case study on the evaluation of discussion and debate learning effectiveness in a dental hygiene ethics class. Eur J Dent Educ.

[33] Moore KG, Clements J, Sease J, Anderson Z (2015). The utility of clinical controversy debates in an ambulatory care elective. Curr Pharm Teach Learn.

[34] Darby M (2007). Debate: a teaching-learning strategy for developing competence in communication and critical thinking. Am Dent Hyg Assoc.

[35] Snider A, Schnurer M (2002). Many sides: Debate across the curriculum: IDEA.

[36] Rudd RD. Defining critical thinking. Techniques: Connecting education and careers. 2007;82(7):46–9.

[37] Dornan T, Conn R, Monaghan H, Kearney G, Gillespie H, Bennett D (2019). Experience based learning (ExBL): clinical teaching for the twenty-first century. Med Teach.

