# Supplementary material for: Debating: effective and satisfactory learning method in dentistry
Source: BMC Med Educ. 2024 Mar 19;24:307. doi: 10.1186/s12909-024-05286-5 (PMC10953255; doi:10.1186/s12909-024-05286-5)
Supplement: Supplementary file 1 — Supplementary Material 1. [file 12909_2024_5286_MOESM1_ESM.docx]

|  | 1 | 2 | 3 | 4 |
| --- | --- | --- | --- | --- |
| **Reasoning:**  Arguments for or against the hypothesis stated | Very little or no real arguments have been made, or all the arguments presented have significant problems. Failure to use scientific studies to support the situation | Some appropriate arguments, but has some basic problems. Failure to use scientific studies to support the situation | Many good arguments are presented, only with minor problems. Moderate use of scientific studies to support the position | Very strong and compelling arguments presented throughout. Good use of scientific studies to support position. |
| **Data interpretation and analysis skills:**  Articulate the main arguments and responses in a clear and orderly manner by providing evidence | Asking enlightening questions. Defending against weak opposing commentators is weak, failing to point out problems if in a positive team position or failing to defend themselves against attack. | Asking enlightening questions and defending against appropriate opposing opinions but with some important problems | Asking good enlightening questions, defending against opposing opinions only with minor slips | Asking excellent mutual enlightenment questions and defending against opposing opinions |
| **Ability to use scientific databases:**  Providing and analyzing reliable scientific resources | Evidence is low or not provided. | Evidence is provided but neither sources are good nor well organized | The evidence is logical and good but not unanimous | Reliable references have been used on key points |
| **Presentation style:**  Use effective verbal and nonverbal communication skills (such as volume, pause, eye contact, etc.) | Very few stylistic features were used, none of them convincing, most of the presentations were read from the text. | A small number of stylistic features were persuasively used, most presentations were read from the text | Most of the style features were convincingly used, only some of the presentations were read from the text. | All features of the style were convincingly used, while reading the presentation from the text was very low. |
| **Critical thinking skills:** identifying weaknesses in the arguments of the opposing team, fully and balanced coverage of the subject and reaching a reliable conclusion at the end of the discussion | Highly incomplete coverage of the subject considerable gap in the presentation of the subject | Complete but unbiased coverage or incomplete but balanced coverage | Generally complete and balanced but stacked, requires more evidence or better sequencing | Full coverage of the subject in each assignment, balanced expression of perspectives |

Appendix1. How to score the performance checklist of the debate teams
